# Antiviral Efficacies of FDA-Approved Drugs against SARS-CoV-2 Infection in Ferrets

**DOI:** 10.1128/mBio.01114-20

**Published:** 2020-05-22

**Authors:** Su-Jin Park, Kwang-Min Yu, Young-Il Kim, Se-Mi Kim, Eun-Ha Kim, Seong-Gyu Kim, Eun Ji Kim, Mark Anthony B. Casel, Rare Rollon, Seung-Gyu Jang, Min-Hyeok Lee, Jae-Hyung Chang, Min-Suk Song, Hye Won Jeong, Younho Choi, Weiqiang Chen, Woo-Jin Shin, Jae U. Jung, Young Ki Choi

**Affiliations:** a Department of Microbiology, Chungbuk National University College of Medicine and Medical Research Institute, Cheongju, Republic of Korea; b Zoonotic Infectious Disease Research Center, Chungbuk National University, Cheongju, Republic of Korea; c Department of Internal Medicine, Chungbuk National University College of Medicine and Medical Research Institute, Cheongju, Republic of Korea; d Department of Molecular Microbiology and Immunology, Keck School of Medicine, University of Southern California, Los Angeles, California, USA; Icahn School of Medicine at Mount Sinai

**Keywords:** COVID-19, severe acute respiratory syndrome coronavirus 2 (SARS-CoV-2), antiviral therapeutics, immunosuppression, serum neutralization, ferrets

## Abstract

Due to the urgent need of a therapeutic treatment for coronavirus (CoV) disease 2019 (COVID-19) patients, a number of FDA-approved/repurposed drugs have been suggested as antiviral candidates at clinics, without sufficient information. Furthermore, there have been extensive debates over antiviral candidates for their effectiveness and safety against severe acute respiratory syndrome CoV 2 (SARS-CoV-2), suggesting that rapid preclinical animal studies are required to identify potential antiviral candidates for human trials. To this end, the antiviral efficacies of lopinavir-ritonavir, hydroxychloroquine sulfate, and emtricitabine-tenofovir for SARS-CoV-2 infection were assessed in the ferret infection model. While the lopinavir-ritonavir-, hydroxychloroquine sulfate-, or emtricitabine-tenofovir-treated group exhibited lower overall clinical scores than the phosphate-buffered saline (PBS)-treated control group, the virus titers in nasal washes, stool specimens, and respiratory tissues were similar between all three antiviral-candidate-treated groups and the PBS-treated control group. Only the emtricitabine-tenofovir-treated group showed lower virus titers in nasal washes at 8 days postinfection (dpi) than the PBS-treated control group. To further explore the effect of immune suppression on viral infection and clinical outcome, ferrets were treated with azathioprine, an immunosuppressive drug. Compared to the PBS-treated control group, azathioprine-immunosuppressed ferrets exhibited a longer period of clinical illness, higher virus titers in nasal turbinate, delayed virus clearance, and significantly lower serum neutralization (SN) antibody titers. Taken together, all antiviral drugs tested marginally reduced the overall clinical scores of infected ferrets but did not significantly affect *in vivo* virus titers. Despite the potential discrepancy of drug efficacies between animals and humans, these preclinical ferret data should be highly informative to future therapeutic treatment of COVID-19 patients.

## Abstract (precis)

The SARS-CoV-2 pandemic continues to spread worldwide, with rapidly increasing numbers of mortalities, placing increasing strain on health care systems. Despite serious public health concerns, no effective vaccines or therapeutics have been approved by regulatory agencies. In this study, we tested the FDA-approved drugs lopinavir-ritonavir, hydroxychloroquine sulfate, and emtricitabine-tenofovir against SARS-CoV-2 infection in a highly susceptible ferret infection model. While most of the drug treatments marginally reduced clinical symptoms, they did not reduce virus titers, with the exception of emtricitabine-tenofovir treatment, which led to diminished virus titers in nasal washes at 8 dpi. Further, the azathioprine-treated immunosuppressed ferrets showed delayed virus clearance and low SN titers, resulting in a prolonged infection. As several FDA-approved or repurposed drugs are being tested as antiviral candidates at clinics without sufficient information, rapid preclinical animal studies should proceed to identify therapeutic drug candidates with strong antiviral potential and high safety prior to a human efficacy trial.

## INTRODUCTION

In December of 2019, a novel coronavirus (CoV) disease (COVID-19), identified in Wuhan, Hubei Province, China, in patients with pneumonia, was found to be caused by a previously unknown *Betacoronavirus*. The outbreak rapidly spread to other provinces in mainland China, and despite great efforts, the epidemic continued to spread from China to Europe, North America, and other Asian countries. The World Health Organization (WHO) announced that the severe acute respiratory syndrome CoV 2 (SARS-CoV-2) epidemic was a public health emergency of international concern on 30 January 2020, and on 11 March 2020, the WHO Director General characterized COVID-19 as a pandemic (http://www.euro.who.int/en/health-topics/health-emergencies/coronavirus-covid-19/news/news/2020/01/2019-ncov-outbreak-is-an-emergency-of-international-concern; https://www.who.int/dg/speeches/detail/who-director-general-s-opening-remarks-at-the-media-briefing-on-covid-19---11-march-2020). Currently, the number of people diagnosed with SARS-CoV-2 infection is increasing by approximately 10,000 cases a day. As of 17 April 2020, approximately 2,074,529 cases had been diagnosed as COVID-19 and 139,378 deaths had occurred (1). Further, recent studies reported the detection of SARS-CoV-2 RNA in various clinical specimens, suggesting other transmission routes for this virus besides respiratory secretions (2–4).

Unfortunately, to date, no vaccines or antiviral drugs have been approved for the treatment of SARS-CoV-2 infection by regulatory agencies. Researchers are fervently working to develop vaccines specifically for this virus, as well as potential treatments for COVID-19. Although many clinical trials are ongoing, there are no specific therapeutic agents approved for COVID-19. Developing SARS-CoV-2-specific antiviral drugs from scratch could take years; drugs that have already been approved by the U.S. Food and Drug Administration (FDA) have the potential to reach patients more quickly. During the SARS outbreak in 2003, screening of approved drugs identified lopinavir-ritonavir, a human immunodeficiency virus type 1 (HIV-1) aspartate protease inhibitor, as effective against SARS-CoV replication (5). The antiviral activity of lopinavir-ritonavir against Middle East respiratory syndrome coronavirus (MERS-CoV) both *in vitro* (6) and in an animal model (7) has been reported, and case reports suggest that the combination of lopinavir-ritonavir with ribavirin and interferon alpha results in virologic clearance and survival (8, 9).

Chloroquine (CQ), a widely used antimalarial with immunomodulatory effects (10), was found in a recent study to inhibit the growth of SARS-CoV-2 *in vitro* (11). However, this finding has not been strongly supported by clinical studies of approximately 100 SARS-CoV-2-infected patients (12, 13). A derivative of chloroquine, hydroxychloroquine (HCQ) sulfate, was first synthesized in 1946 by adding a hydroxyl group to CQ, resulting in a compound found to be much less toxic than CQ in an animal study (14). In autoimmune diseases, HCQ sulfate works by reducing inflammation (15). However, recent reports have also shown heart risk concerns with the use of CQ and HCQ sulfate for COVID-19 treatment.

Emtricitabine-tenofovir (Truvada) is a prescription medicine for HIV approved by the U.S. FDA for preexposure prophylaxis to reduce the risk of HIV infection in adults and adolescents. As a nucleotide analogue, it is reported that the active triphosphate form of this tenofovir diphosphate inhibits activity for RNA-dependent RNA polymerase (RdRp) of HIV and hepatitis B virus (HBV) (16, 17). Still, even these existing drugs will need rigorous testing for efficacy and safety and ultimately ramped-up production before they can be deployed widely against COVID-19.

Generally, immunocompromised patients are more susceptible to bacterial, fungal, viral, and parasitic infections than healthy persons due to their inability to mount successful immune responses. This can be caused by impairment or weakening of the immune system by a number of conditions, including diseases (e.g., diabetes or HIV infection), malnutrition, and the use of certain medications. It has become apparent that SARS-CoV-2 infection also affects immunocompromised individuals more severely. A majority of COVID-19 patients who were clinically diagnosed are older than ∼60 years and have underlying complications, including heart disease, diabetes, hypertension, or cancer, indicating that age and reduced immune activity are the critical risk factors or determinants for COVID-19 morbidity and mortality.

We have recently established a ferret model for SARS-CoV-2 infection and transmission that highly recapitulates aspects of the human infection (18). Elevated body temperatures and virus replication were readily detected in SARS-CoV-2-infected ferrets. SARS-CoV-2-infected ferrets shed the virus through nasal washes and in saliva, urine, and fecal specimens. SARS-CoV-2 was transmitted readily to naive direct-contact ferrets but less efficiently to naive indirect-contact ferrets (18). Further, acute bronchiolitis was observed in infected lungs. In this report, we evaluated the efficacy of oral administration of lopinavir-ritonavir, HCQ sulfate, and emtricitabine-tenofovir for SARS-CoV-2 infection in ferret infection models. We also treated ferrets with azathioprine, an immunosuppressive drug, and evaluated the replication kinetics of SARS-CoV-2. While most drug treatments reduced clinical symptoms (CS), none of them led to a significant reduction of *in vivo* virus titers in ferrets. Thus, a drug candidate study in a robust preclinical animal model should greatly facilitate testing the efficacies and safety of therapeutic treatments for COVID-19 patients.

## RESULTS

### Clinical features of SARS-CoV-2-inoculated ferrets treated with antivirals.

**FIG 1:**
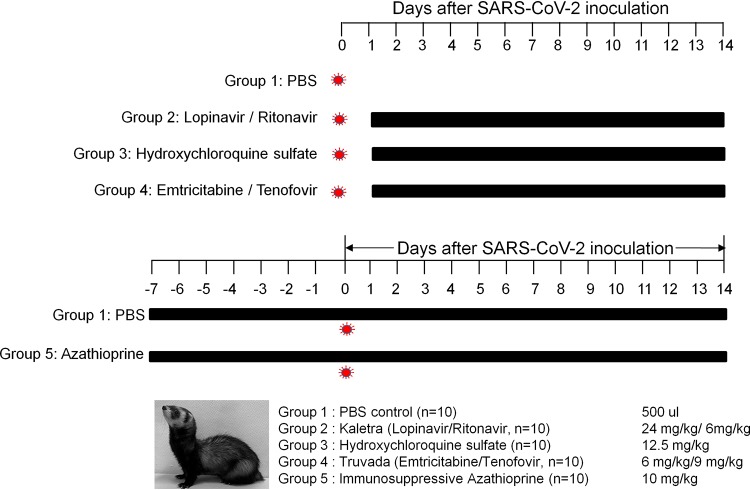
Schedule of drug treatments and SARS-CoV-2 infection in ferrets. To induce the immunosuppression condition, PBS or azathioprine was orally administered to ferrets for the entire experimental period. All groups of ferrets were administered each drug or PBS via oral gavage once, starting at 1 dpi.

**FIG 2:**
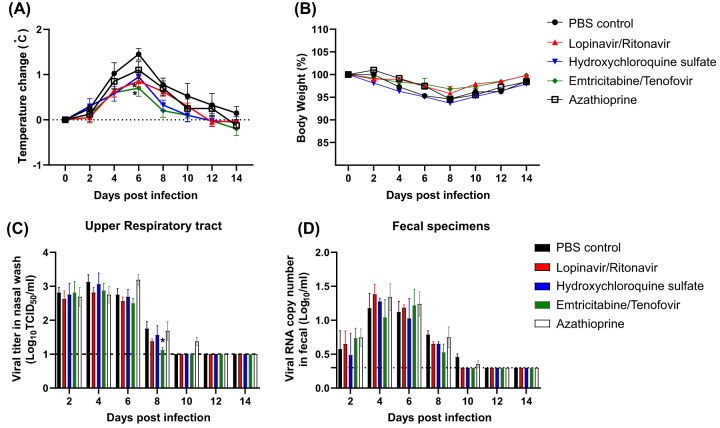
Clinical features of drug-treated ferret groups. Ten ferrets per group were inoculated via the i.n. route with 10^5.8^ TCID_50_ of the NMC-nCoV02 strain. Starting at 1 day of postinfection, lopinavir-ritonavir, hydroxychloroquine sulfate, emtricitabine-tenofovir, or azathioprine was orally administered to each group. Temperature changes (A) and relative weight changes (B) are shown with standard errors of the means. (A) Temperature is represented as relative change (degrees Celsius), and (B) weight change is demonstrated as a percentage of the initial body weight. Nasal wash and fecal samples were collected at 2, 4, 6, 8, 10, 12, and 14 dpi from nasal wash specimens and fecal swabs. (C) Virus titers (TCID_50_) were measured in nasal wash specimens from each group. (D) The number of viral RNA copies was measured in fecal samples using qRT-PCR. Viral titers and RNA copy numbers are shown as means ± standard errors of the means (SEM) from four animals, and titers below the limit of detection are shown as 1 log_10_ TCID_50_/ml or 0.3 log_10_ viral RNA copy numbers/ml (dashed lines). Asterisks indicate statistical significance between the control and each group as determined by two-way ANOVA and subsequent Dunnett’s test (*, *P < *0.05).

In order to determine the antiviral efficacies of lopinavir-ritonavir, hydroxychloroquine (HCQ) sulfate, or emtricitabine-tenofovir for treatment of SARS-CoV-2 infection, SARS-CoV-2 antibody-free ferrets (10/group) were inoculated with 10^5.8^ 50% tissue culture infective doses (TCID_50_)/ml of an NMC-nCoV02 strain through the intranasal (i.n.) route (Fig. 1). At 1 day postinfection (dpi) with SARS-CoV-2, ferrets were administered lopinavir (24 mg/kg of body weight)-ritonavir (6 mg/kg), hydroxychloroquine sulfate (12.5 mg/kg), or emtricitabine (6 mg/kg)-tenofovir (9 mg/kg) daily via oral gavage for 14 days (Fig. 1). In addition, to test the effect of immunosuppression on viral infection and clinical outcome, a group (*n* = 10) of ferrets was also treated with phosphate-buffered saline (PBS) (as a control) or azathioprine, an immunosuppressive drug (10 mg/kg), for 7 days prior to SARS-CoV-2 infection (Fig. 1). While all groups of SARS-CoV-2-infected ferrets showed elevated temperatures at 4 to 6 dpi, the lopinavir-ritonavir- or emtricitabine-tenofovir-treated group exhibited mild fever compared with the PBS-treated group (Fig. 2A). As with the PBS-treated group, the hydroxychloroquine sulfate- or azathioprine-treated group showed ∼7% body weight loss at 8 dpi, while the lopinavir-ritonavir- or emtricitabine-tenofovir-treated group showed a <4% change in body weight on average (Fig. 2B). To compare clinical features of SARS-CoV-2 infection following treatment with each drug, we developed an arbitrary scoring method to generate clinical symptom values based on 20-min observations of cough, rhinorrhea, and reduced activity and compared these CS values among the ferret groups as described in Table 1. The average CS value of PBS-treated control ferrets was 3.5 at 2 dpi, remained relatively high (>5) for 3 to 6 dpi, and returned to normal (less than 1) by 11 dpi (Table 1). The lopinavir-ritonavir-treated ferrets showed the reduced overall CS values (less than 5) that peaked at 4 to 5 dpi and resolved by 10 dpi (Table 1). The HCQ sulfate-treated ferrets also showed reduced CS values at 6 to 8 dpi compared with those of the PBS-treated group, but overall clinical symptoms were similar to those of the PBS-treated control group (Table 1). Although the emtricitabine-tenofovir-treated ferrets initially demonstrated clinical symptoms similar to those of the PBS-treated control group, their overall CS values were relatively low at 3 to 8 dpi, and clinical symptoms were ultimately resolved by 9 dpi. Finally, the azathioprine-treated immunosuppressed ferrets also showed CS values similar to those of the PBS-treated control group, but this group’s clinical symptoms lasted slightly longer than those of the PBS-treated control group (Table 1). These results collectively showed that the lopinavir-ritonavir-, HCQ sulfate-, or emtricitabine-tenofovir-treated group exhibited lower overall clinical scores than the PBS-treated control group. The overall clinical symptoms of immunosuppressed ferrets were similar but persisted slightly longer than those of PBS-treated control ferrets.

**TABLE 1:** Clinical scores of ferrets treated with each candidate antiviral drug^*a*^

Group and symptom	Score at day:												
	0	1	2	3	4	5	6	7	8	9	10	11	14
PBS control													
Cough	0.00	0.25 ± 0.43	1.00	1.50 ± 0.50	1.75 ± 0.43	1.50 ± 0.50	1.25 ± 0.43	1.00	1.00	0.50 ± 0.50	0.00	0.00	0.00
Rhinorrhea	0.00	0.50 ± 0.50	1.00 ± 0.71	1.50 ± 0.50	2.00	2.00	2.00	1.50 ± 0.50	1.00	1.00	1.00	0.00	0.00
MA	0.00	0.75 ± 0.43	1.50 ± 0.50	2.00	2.00	2.00	2.00	1.50 ± 0.50	1.00	1.00	0.50 ± 0.50	0.00	0.00
Total	0.00	1.50	3.50	5.00	5.75	5.50	5.25	4.00	3.00	2.50	1.50	0.00	0.00

Lopinavir-ritonavir													
Cough	0.00	0.00	0.25 ± 0.43	0.50 ± 0.50	1.00	1.00	1.00	0.75 ± 0.43	0.00	0.00	0.00	0.00	0.00
Rhinorrhea	0.00	0.00	1.00	1.50 ± 0.50	2.00	2.00	1.75 ± 0.43	1.25 ± 0.43	1.00	0.50 ± 0.50	0.00	0.00	0.00
MA	0.00	0.75 ± 0.43	1.00	1.75 ± 0.43	1.75 ± 0.43	1.75 ± 0.43	1.25 ± 0.43	1.00	1.00	1.00	0.75 ± 0.43	0.00	0.00
Total	0.00	0.75	2.25	3.75	4.75	4.75	4.00	3.00	2.00	1.50	0.75	0.00	0.00

Hydroxychloroquine sulfate													
Cough	0.00	0.00	0.75 ± 0.43	1.00	1.00	1.00	0.75 ± 0.43	0.50 ± 0.50	0.00	0.00	0.00	0.00	0.00
Rhinorrhea	0.00	0.25 ± 0.43	1.25 ± 0.43	1.75 ± 0.43	2.00	2.00	1.25 ± 0.43	1.25 ± 0.43	1.00	1.00	0.50 ± 0.50	0.00	0.00
MA	0.00	0.50 ± 0.50	1.50 ± 0.50	2.00	2.00	1.75 ± 0.43	1.50 ± 0.50	1.00	1.00	1.00	0.50 ± 0.50	0.00	0.00
Total	0.00	0.75	3.50	4.75	5.00	4.75	3.50	2.75	2.00	2.00	1.00	0.00	0.00

Emtricitabine- tenofovir													
Cough	0.00	0.50 ± 0.50	0.75 ± 0.43	1.00	0.50 ± 0.50	0.50 ± 0.50	0.00	0.00	0.00	0.00	0.00	0.00	0.00
Rhinorrhea	0.00	0.75 ± 0.43	1.00	1.00	1.75 ± 0.43	2.00	1.25 ± 0.43	1.00	0.50 ± 0.50	0.00	0.00	0.00	0.00
MA	0.00	1.00	1.00	1.00	1.25 ± 0.43	1.25 ± 0.43	1.00	1.00	0.75 ± 0.43	0.25 ± 0.43	0.00	0.00	0.00
Total	0.00	2.25	2.75	3.00	3.50	3.75	2.25	2.00	1.25	0.25	0.00	0.00	0.00

Azathioprine													
Cough	0.00	0.00	0.75 ± 0.43	1.00	1.00	1.00	1.00	1.00	0.50 ± 0.50	0.00	0.00	0.00	0.00
Rhinorrhea	0.00	0.25 ± 0.43	1.00	1.75 ± 0.43	1.75 ± 0.43	1.75 ± 0.43	1.50 ± 0.50	1.00	1.00	0.75 ± 0.43	0.50 ± 0.50	0.00	0.00
MA	0.00	1.00	1.00	1.25 ± 0.43	1.75 ± 0.43	1.75 ± 0.43	1.75 ± 0.43	1.50 ± 0.50	1.00	1.00	1.00	1.00	0.00
Total	0.00	1.25	2.75	4.00	4.50	4.50	4.25	3.50	2.50	1.75	1.50	1.00	0.00

a Observational clinical symptoms were cough, rhinorrhea, and movement and activity (MA). Scores: 0, normal; 1, occasional or mildly reduced activity; 2, frequently reduced activity. Scores were measured by clinical observation of symptoms for at least 20 min for each group of ferrets on the basis of the following criteria. For cough, 0, no evidence of cough; 1, occasional cough; 2, frequent cough (score 2). For rhinorrhea, 0, no nasal rattling or sneezing; 1, moderate nasal discharge on external nares; 2, severe nasal discharge on external nares. For movement and activity, 0, normal movement and activity; 1, mild reduced movement and activity; 2, evidence of reduced movement and activity.

### Comparisons of virus titers and shedding periods in antiviral-drug-treated ferrets.

To evaluate the antiviral activity of each drug against SARS-CoV-2, we measured infectious virus titers in nasal washes from drug-treated ferrets (Fig. 2C). SARS-CoV-2 was isolated from all infected ferrets regardless of drug treatment from 2 dpi to 6 dpi, with similar virus titers (2.75 to 3.2 log_10_ TCID_50_/ml). At 8 dpi, the emtricitabine-tenofovir-treated ferrets exhibited reduced virus titers compared with those of the PBS-treated control group. Although infectious virus was not detected in ferrets of the PBS- or antiviral-drug-treated groups at 10 dpi, three of four azathioprine-treated ferrets were positive for virus even at 10 dpi (Fig. 2C), suggesting delayed virus clearance in the upper respiratory tracts of immunocompromised ferrets.

Because gastrointestinal involvement has been documented in coronavirus infections of animals and humans (19), we also collected fecal specimens and performed quantitative real-time PCR (qRT-PCR) to determine whether any of the drug treatments affected SARS-CoV-2 shedding in the gastrointestinal system (Fig. 2D). The results showed that the viral RNA was present in fecal specimens of all groups from 2 to 8 dpi, with peak viral RNA copy numbers observed at 4 to 6 dpi. However, there was no statistical difference in viral RNA copy numbers among the groups during the experimental period. By 10 dpi, viral RNA copy numbers declined in all drug-treated ferrets.

**FIG 3:**
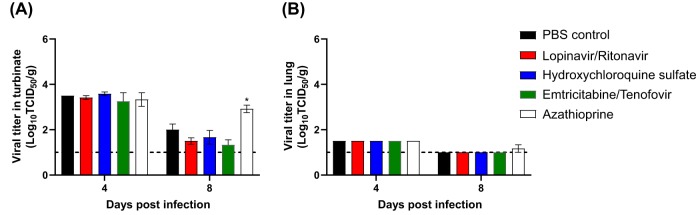
Virus titers in respiratory tissues. Groups of ferrets (3/group) were sacrificed at 4 and 8 dpi. Viral titers were measured in turbinate (A) and lung (B) by determining the numbers of TCID_50_ per gram. The limit of detection is 1 log_10_ TCID_50_/g and indicated by the dotted line for each representation.

To further evaluate virus titers in tissues, three ferrets from each group were euthanized at 4 and 8 dpi, and virus titers were measured in nasal turbinate and lungs. At 4 dpi, all groups of ferrets showed high virus titers of more than 3.0 log_10_ TCID_50_/g in nasal turbinate tissues, and the virus was also isolated from their lung tissues (Fig. 3). At 8 dpi, while all nasal turbinate tissues were positive for virus, the azathioprine-treated group showed a much higher virus titer (∼3.0 log_10_ TCID_50_/g) than those of the other groups (Fig. 3A). At 8 dpi, the azathioprine-treated group still had detectable virus titers in their lung tissues, whereas the rest of groups were negative for the virus (Fig. 3B).

### SARS-CoV-2 antibody neutralization titers in antiviral-drug-treated ferrets.

**FIG 4:**
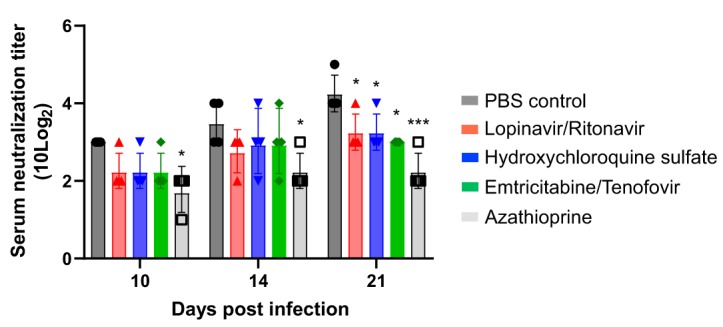
Comparison of serum neutralization antibody titers of drug-treated ferrets. Blood was collected at 10, 14, and 21 dpi from each group of ferrets (*n* = 4), and serum neutralization antibody titers were measured in Vero cells. The serum neutralization titer of each ferret is represented by an individual dot in each bar graph. Asterisks indicate statistical significance between the control and each group, as determined by two-way ANOVA and subsequent Dunnett’s test (*, *P < *0.05; ***, *P < *0.001).

To compare the serum neutralization (SN) antibody titers among drug-treated groups, blood was collected from each group of ferrets at 10, 14, and 21 dpi. At 10 dpi, the PBS-treated control and drug-treated groups demonstrated SN titers greater than 40 (Fig. 4). The antiviral-treated groups showed SN titers similar to those of the PBS-treated control group until 14 dpi, but they exhibited lower SN titers at 21 dpi than the PBS-treated control group (Fig. 4). It is noteworthy that the azathioprine-treated immunosuppression group showed geometric mean SN titers of 33.6 and 47.5 at 10 and 21 dpi, respectively, suggesting the continuously reduced SN antibody response of the immunosuppressed group.

## DISCUSSION

In this study, we evaluated the antiviral efficacies of three FDA-approved drug candidates against SARS-CoV-2 infection using a ferret infection model which has previously proven to be highly susceptible to SARS-CoV-2 infection (18, 20). Although several clinical trials continue to evaluate these drug candidates, most of the enrolled patient populations are considered heterogeneous with regard to the duration and severity of illness at enrollment. Further, given the rapid spread of COVID-19 around the world, there are relatively higher mortality rates in some regions and in certain age groups. Therefore, the use of highly susceptible animal models and controlled experimental settings should be an effective approach prior to human clinical trials to evaluate the *in vivo* antiviral effects of potential therapeutics.

Ferrets treated with antivirals showed relatively reduced overall clinical scores compared with those of the PBS-treated control group, which displayed high overall clinical scores. Of the three drugs, the emtricitabine-tenofovir-treated group showed a noticeable reduction in overall clinical scores (≤4) and a shorter duration (8 dpi) of clinical symptoms. Although attenuation of the overall clinical scores was observed in lopinavir-ritonavir- and HCQ-treated groups at some time points, their clinical durations were comparable with those of the PBS-treated control group. These results suggest that treatment with emtricitabine-tenofovir, a nucleotide analogue that inhibits RNA-dependent RNA polymerase activity, may be the most likely candidate to reduce clinical symptoms, including the cough and morbidity of SARS-CoV-2-infected hosts. While the lopinavir-ritonavir protease inhibitor and a CQ/HCQ sulfate autophagy inhibitor have been shown to be effective against SARS-CoV, MERS-CoV, or SARS-CoV-2 in *in vitro* culture (5), several reports have already described no benefit for clinical improvement of COVID-19 patients. Furthermore, HQC has been reported to be associated with a number of side effects, including a heart rhythm problem, severely low blood pressure, and muscle or nerve damage. Thus, these existing FDA-approved drugs still need rigorous testing for efficacy and safety in an animal model prior to clinical trials of COVID-19 patients.

Since the emergence of SARS-CoV-2 in China in December 2019, the number of cases has rapidly increased as the disease has spread globally. The increase in the number of cases is alarming and specially compounded by the possibility of viral transmission from asymptomatic individuals. Several studies indicate that asymptomatic patients can transmit the virus to persons in close contact (21, 22). Therefore, although clinical symptoms were attenuated in ferret groups treated with antiviral candidates, we also evaluated virus titers in respiratory and gastrointestinal tracts using nasal washes and stool samples, respectively, from SARS-CoV-2-infected ferrets. Regardless of antiviral candidate treatment, SARS-CoV-2 was detected at more than 2 log_10_ TCID_50_/ml until 6 dpi, and there was no statistical difference between the PBS-treated control group and the antiviral-drug-treated groups. However, the emtricitabine-tenofovir-treated group showed relatively low virus tiers and shortened periods of virus shedding in nasal wash specimens compared with those of the other groups. Currently, the role of SN antibody in the pathogenesis and disease clearance of SARS-CoV-2 is unclear. Wu et al. (23) recently reported that the SN antibody levels in COVID-19 patients were variable, depending on the immune status of the patient, and that about 30% of patients failed to develop high SN titers after SARS-CoV-2 infection, although the disease durations of these patients were comparable to those of others. This suggests that the SN antibody titer may be closely associated with immune activity rather than with the virus titer and disease duration in patients. Interestingly, we found that the antiviral-treated groups showed lower SN antibody titers than the PBS-treated control group. It is possible that as the antiviral treatment reduced the overall clinical symptoms of SARS-CoV-2-infected ferrets, it might evoke weak immune responses and thereby lead to reduced neutralizing antibody responses. Nevertheless, further immunological studies are needed to understand the detailed mechanisms of the low SN antibody titers in antiviral-treated ferrets.

While COVID-19 is typically characterized by respiratory symptoms, gastrointestinal symptoms have been reported in some cases. Moreover, there is evidence of viral RNA in the stools of SARS-CoV-2 patients. Emtricitabine-tenofovir has reportedly shown antiviral efficacy in the gastrointestinal tract as well as in the respiratory tract (24). However, qRT-PCR analysis of stools revealed no statistical difference in virus titers in stools among the PBS-treated control and the antiviral-treated groups, suggesting that none of the tested antiviral candidates significantly diminished gastrointestinal SARS-CoV-2 replication in infected ferrets. In conclusion, although there may be some discrepancies in drug efficacy between animals and humans, these results of a preclinical ferret infection study should aid in the selection of antiviral treatments of COVID-19 patients. This also suggests that a robust preclinical animal model for SARS-CoV-2 infection is valuable in order to identify antiviral drugs for future human efficacy trials.

## MATERIALS AND METHODS

### Virus and cells.

A SARS-CoV-2 strain, NMC-nCoV02, was propagated in Vero cells in Dulbecco’s modified Eagle medium (DMEM; Gibco, Grand Island, NY) supplemented with 1% penicillin-streptomycin (Gibco) and TPCK (tosylsulfonyl phenylalanyl chloromethyl ketone)-treated trypsin (0.5 μg/ml; Worthington Biochemical, Lakewood, NJ) in a 37°C incubator supplemented with 5% CO_2_ for 72 h. Propagated virus was stored at −80°C as the working virus stock for animal studies. The 50% tissue culture infective dose (TCID_50_) was determined through fixation and crystal violet staining.

### Immunosuppression and antiviral-drug candidate treatments.

Ten ferrets were treated orally with azathioprine (10 mg/kg) (Celltrion) daily for 7 days prior to SARS-CoV-2 infection, and treatment continued until 14 days postinfection (dpi) to reduce the immune response. As a control, PBS in the same volume was administered to 10 ferrets. To confirm the immunosuppressed status of ferrets, blood samples were collected from azathioprine-treated ferrets, and the percentage of lymphocytes was assessed at 7 and 4 days preinfection and at 7, 14, and 21 dpi. The reduction in lymphocyte numbers in azathioprine-treated ferrets compared with lymphocyte numbers in the PBS control group was confirmed (see Fig. S1 in the supplemental material). For treatment with candidate antiviral drugs, groups of ferrets (10/group) were administered lopinavir (16 mg/kg)-ritonavir (4 mg/kg) (Abbott), hydroxychloroquine sulfate (25 mg/kg) (Elyson), or emtricitabine (6 mg/kg)-tenofovir (7.35 mg/kg) (Gilead) daily via oral gavage starting at 1 dpi of SARS-CoV-2 infection and continuing until 14 dpi.

10.1128/mBio.01114-20.1 FIG S1 Lymphocyte counts in ferrets after treatment with azathioprine or PBS. Ten ferrets were orally administered azathioprine (10 mg/kg) or PBS. Blood was collected from azathioprine-treated ferrets, and their lymphocyte numbers were measured using hematological parameters and the Celltac hematology analyzer (MEK-6550J/K; Nihon Kohden). Download FIG S1, PDF file, 0.1 MB. Copyright © 2020 Park et al. 2020 Park et al. This content is distributed under the terms of the Creative Commons Attribution 4.0 International license.

### Experimental infection of ferrets.

Groups of 10- to 12-month-old female ferrets (10/group), seronegative for SARS-CoV-1 and SARS-CoV-2, were intranasally inoculated with 10^5.8^ TCID_50_/ml of NMC-nCoV02 under anesthesia. The body weights and temperatures of infected ferrets were monitored every other day until 14 dpi. Nasal washes and stool specimens were collected every other day from the inoculated ferrets. Blood was collected at 10, 14, and 21 dpi to measure the serum neutralization titer. Three ferrets per group were euthanized at 4 and 8 dpi, and nasal turbinate and lungs were collected to measure tissue virus titers and examine lung histopathology. Virus titers in nasal washes and tissues were determined by 50% TCID_50_ assessment in Vero cells, while the virus titers in stool specimens were measured with quantitative real-time PCR (qRT-PCR). Briefly, total RNA was extracted using TRIzol reagent (Thermo Fisher Scientific) or an RNeasy kit (Qiagen), and cDNAs were generated with a SARS-CoV-2-specific primer by reverse transcription using QuantiTect reverse transcription (Qiagen). qRT-PCRs were performed using a SYBR green supermix (Bio-Rad) and a CFX96 Touch real-time PCR detection system (Bio-Rad) with a spike gene-based, SARS-CoV-2-specific primer set as previously described (18), and virus RNA copy numbers were calculated as a ratio with respect to the standard control.

### Clinical scoring methods of SARS-CoV-2-infected ferrets.

The ferrets were monitored daily over a 14-day period for temperature change, weight loss, clinical symptom, and movement and activity change. Briefly, the frequency of cough and rhinorrhea was assessed in each group of ferrets and scored on the basis of the following criteria: no evidence of cough (score, 0), occasional cough (score, 1), and frequent cough (score, 2) and no nasal rattling or sneezing (score, 0), moderate nasal discharge on external nares (score, 1), and severe nasal discharge on external nares (score, 2). A change in a ferret’s activity was assessed and scored on the basis of the following criteria: normal movement and activity (score, 0), mildly reduced movement and activity (score, 1), and considerably reduced movement and activity (score, 2) for at least 20 min.

### Neutralizing assay.

Sera were collected from each group of ferrets to detect the serum neutralization titer. Heat-inactivated 10-fold-diluted serum samples were serially diluted by 2-fold. An equal volume of SARS-CoV-2 at 100 TCID_50_ was added to all diluted samples. The mixture of serum and virus was incubated at 37°C for 1 h and then added to Vero cells in a 96-well tissue culture plate for 90 min. The mixture of serum and virus was then removed, followed by two washes with cold PBS. Fresh medium was added to infected cells, and cells were incubated at 37°C in 5% CO_2_ for 4 days. Supernatants were removed, fixed with a 10% formalin solution, and stained with crystal violet to determine the titer.

### Statistical analysis.

To assess significant differences in values for weight loss, temperature, viral titers, and serum neutralization titers, statistical analyses were done. Asterisks indicate the statistical significance between PBS-administered and treated ferrets determined by two-way analysis of variance (ANOVA) and a subsequent Dunnett test (*, *P < *0.05; **, *P < *0.001; and ***, *P < *0.0001). All statistical analyses were performed using GraphPad Prism version 8.20 for Windows (GraphPad Software, La Jolla, CA).

### Ethics statement.

All animal experiments were approved by the Medical Research Institute, a member of the Laboratory Animal Research Center of Chungbuk National University (LARC) (approval number CBNUR-1352-20), and were conducted in strict accordance with and adherence to relevant policies regarding animal handling as mandated under the Guidelines for Animal Use and Care of the Korea Centers for Disease Control (KCDC). The handling of virus was performed in an enhanced biosafety level 3 (BSL3) containment laboratory as approved by the Korea Centers for Disease Control and Prevention (protocol KCDC-14-3-07).
