# Supplementary material for: Antiviral Efficacies of FDA-Approved Drugs against SARS-CoV-2 Infection in Ferrets
Source: mBio. 2020 May 22;11(3):e01114-20. doi: 10.1128/mBio.01114-20 (PMC7244896; doi:10.1128/mBio.01114-20)
Supplement: FIG S1 [file mBio.01114-20-sf001.pdf]

Supplemental Materials

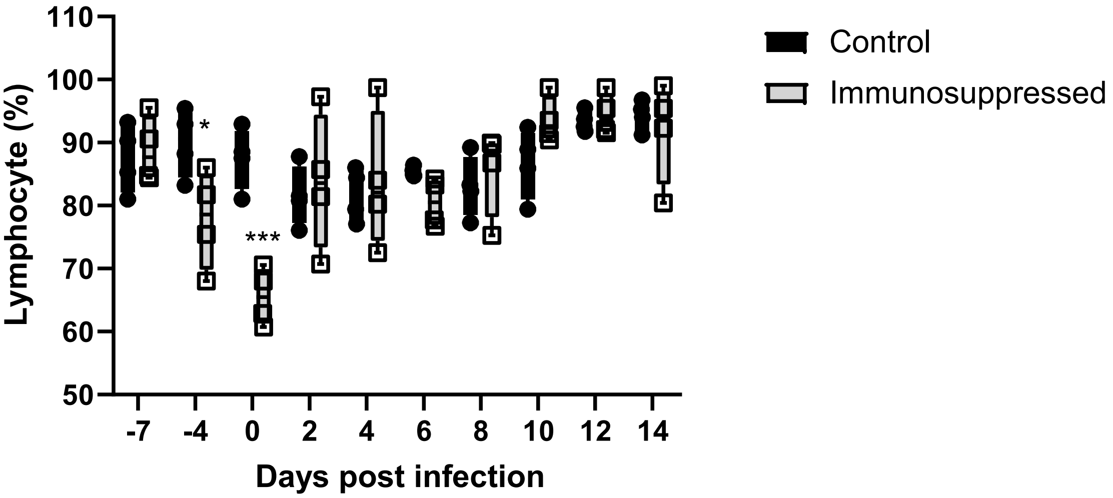

Figure S1. Lymphocyte counts in ferrets after treatment with azathioprine or PBS. Ten ferrets were orally administered azathioprine (10 mg/kg) or PBS. Blood was collected from azathioprine-treated ferrets and their lymphocyte numbers were measured using hematological parameters and the Celltac hematology analyzer (MEK-6550J/K, Nihon Kohden).
